# Supplementary material for: Modular control of vertebrate axis segmentation in time and space
Source: EMBO J. 2024 Aug 9;43(18):4068–91. doi: 10.1038/s44318-024-00186-2 (PMC11405765; doi:10.1038/s44318-024-00186-2)
Supplement: Supplementary file 1 — Data EV1 [file 44318_2024_186_MOESM1_ESM.zip › Data EV1.docx]

Data EV1.

custom-made Fiji macro-script for concatenating and extracting fluorescent intensity measurements from a defined ROI.
