# Supplementary material for: Modular control of vertebrate axis segmentation in time and space
Source: EMBO J. 2024 Aug 9;43(18):4068–91. doi: 10.1038/s44318-024-00186-2 (PMC11405765; doi:10.1038/s44318-024-00186-2)
Supplement: Supplementary file 1 — Data EV1 [file 44318_2024_186_MOESM1_ESM.zip › Data_EV1_TrackingROI.txt]

path0 = File.openDialog("Select the BF image path");dir0 = File.getParent(path0);name0 = File.getName(path0);basename = substring(name0,0,lengthOf(name0)-5);for (l=1; l<=11; l++) {      j = l;      print(j);if (l==10){	basename = substring(basename,0,lengthOf(basename)-1);}open(dir0 +"/"+basename+l+".tif");roiManager("Open", dir0 +"/"+basename+l+".zip");roiManager("Sort");roiManager("List");selectWindow("Overlay Elements of "+basename+l+".tif");table = getInfo("window.contents");ROIs = split(table, "\n");ROIline=split(ROIs[ROIs.length-1], "\t");time=(ROIline[14]);diameter=(ROIline[6]);XArray=newArray(time);YArray=newArray(time);for (i=1; i<(ROIs.length-1); i++) {ROIline1=split(ROIs[i], "\t");x1U=(ROIline1[4]);y1U=(ROIline1[5]);t1U=(ROIline1[14]);XArray[(t1U-1)]=x1U;YArray[(t1U-1)]=y1U;ROIline2=split(ROIs[i+1], "\t");x2U=(ROIline2[4]);y2U=(ROIline2[5]);t2U=(ROIline2[14]);dx=(x2U-x1U)/(t2U-t1U);dy=(y2U-y1U)/(t2U-t1U);	for (k=1; k<(t2U-t1U); k++) {	XArray[(t1U-1+k)]=x1U+(k*dx);	YArray[(t1U-1+k)]=y1U+(k*dy);	}}ROIline1=split(ROIs[ROIs.length-1], "\t");xLU=(ROIline1[4]);yLU=(ROIline1[5]);tLU=(ROIline1[14]);XArray[(tLU-1)]=xLU;YArray[(tLU-1)]=yLU;//roiManager("Save", dir0+"/"+substring(name0,0,lengthOf(name0)-4)+".zip");selectWindow(basename+l+".tif");Stack.setPosition(1, 1, 1); //(channel, slice, frame)k = newArray();//loop over frames	for (m=1; m<=tLU; m++) {		Stack.setFrame(m);	//make selection circle	run("Overlay Options...", "stroke=cyan width=1 fill=none set");	makeOval(XArray[m-1],YArray[m-1],diameter,diameter);	run("Measure");	k = Array.concat(k,getResult("Mean"));	run("Add Selection...");}run("Clear Results");for (j=0; j<k.length; j++) {  setResult("Value", j, k[j]); }updateResults();saveAs("Measurements",dir0 +"/"+basename+l+".csv");roiManager("Deselect");roiManager("Delete");selectWindow("Overlay Elements of "+basename+l+".tif");run("Close");selectWindow("Results");run("Close");saveAs("Tiff", dir0+"/overlayed/"+name0);close();}
