# Supplementary material for: Modular control of vertebrate axis segmentation in time and space
Source: EMBO J. 2024 Aug 9;43(18):4068–91. doi: 10.1038/s44318-024-00186-2 (PMC11405765; doi:10.1038/s44318-024-00186-2)
Supplement: Supplementary file 2 — Data EV2 [file 44318_2024_186_MOESM2_ESM.zip › Data EV2.docx]

Data EV2.

VEP analysis for segmentation timing hits
