# Supplementary material for: Modular control of vertebrate axis segmentation in time and space
Source: EMBO J. 2024 Aug 9;43(18):4068–91. doi: 10.1038/s44318-024-00186-2 (PMC11405765; doi:10.1038/s44318-024-00186-2)
Supplement: Supplementary file 5 — Data EV5 [file 44318_2024_186_MOESM5_ESM.zip › Data EV5.docx]

Data EV5.

VEP analysis for PSM size hits
