# Supplementary material for: Modular control of vertebrate axis segmentation in time and space
Source: EMBO J. 2024 Aug 9;43(18):4068–91. doi: 10.1038/s44318-024-00186-2 (PMC11405765; doi:10.1038/s44318-024-00186-2)
Supplement: Supplementary file 7 — Appendix [file 44318_2024_186_MOESM7_ESM.pdf]

Appendix Table of Contents

Appendix Figure S1. her7-venus period measurements in Hybrid F1 populations......................3

Appendix Figure S2. F2 Kaga/Cab her7-venus reveals modular nature of segmentation timing and unsegmented PSM/somite size 5

Appendix Figure S3. Whole genome sequencing of F0, F1 and F2 Kaga/Cab 7

Appendix Figure S4. Functional validation of devQTL hits using CRISPR/Cas9 KOs segmentation timing..9

Appendix Figure S5. Functional validation of devQTLs on unsegmented PSM size using F0 Crispants and HCR 11

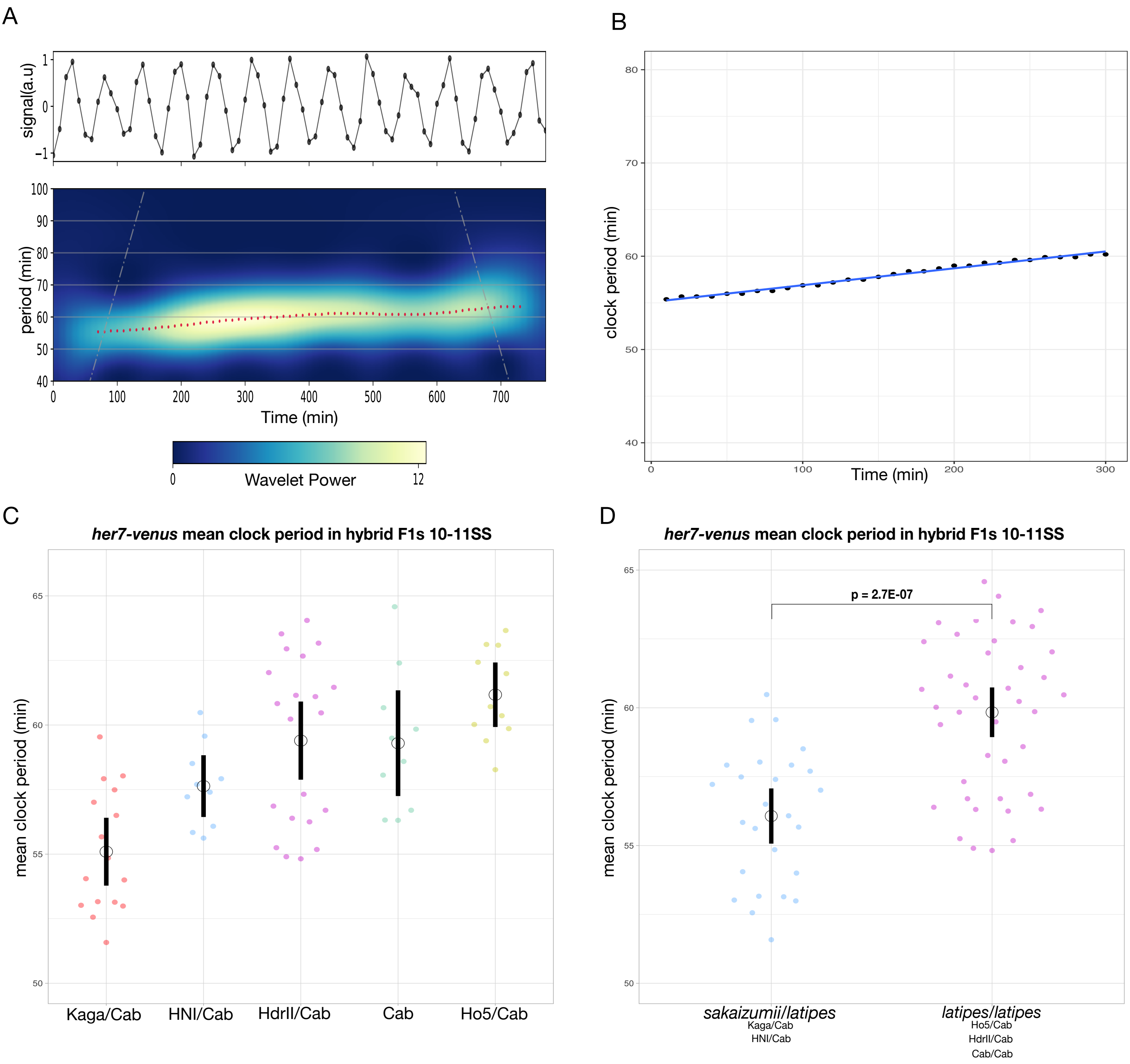

Seleit et al., Supplementary Figure 1

Appendix Figure S1. *her7-venus* period measurements in Hybrid F1 populations

(A) PyBoat wavelet transformation of detrended signal from Figure 2C shows continuous instantaneous period measurements over the course of live-imaging (B) example of period measurements every with 10 minute intervals for 300 minutes from the wavelet transformation in (A). Individual period measurements are black dots with linear-fit in blue. Mean period is the average of the black dot period measurements over 300 minutes, intercept period (clock period) is the y-intercept value of the blue fitted line. Period in minutes, Time in minutes. (C) endogenous *her7-venus* mean period measurements in hybrid F1 embryos at the 10-11SS. Kaga/Cab F1 hybrids have the fastest mean *her7-venus* period (55.09 minutes (sd +/- 2.39)), while HNI/Cab F1s show (57.63 minutes (sd +/- 1.59)), on the other hand hybrid F1 *O.latipes* strains Cab/Cab show (59.3 minutes (sd +/- 2.71)), Cab/HdrII show (59.4 minutes (sd +/- 3.24)), Cab/Ho5 show (61.17 minutes (sd +/-1,77)). Black circle = mean Black line = 95% confidence interval. One-way ANOVA p= 1.2E-06. Post-Hoc Tukey HSD testing shows significant differences between the following groups Kaga/Cab and Cab/Cab p adjusted = 1.2E-03, Kaga/Cab and HdrII/Cab p adjusted = 4.0E-05, Kaga/Cab and Ho5/Cab p adjusted = 9.0E-07, HNI/Cab and Ho5/Cab p adjusted = 2.1E-03. N= 16 Kaga/Cab F1, N= 10 HNI/Cab F1, N= 10 Cab/Cab F1, N= 21 HdrII/Cab F1, N= 11 Ho5/Cab F1. N= 16 Kaga/Cab F1, N= 10 HNI/Cab F1, N= 10 Cab/Cab F1, N= 21 HdrII/Cab F1, N= 11 Ho5/Cab F1. (D) endogenous *her7-venus* mean period measurements in hybrid F1 of *Oryzias sakaizumii/Oryzias latipes* or *Oryzias latipes/Oryzias latipes* embryos at the 10-11SS. *Oryzias sakaizumii/Oryzias latipes* F1 hybrids have a faster mean *her7-venus* period (56.07 minutes (sd +/- 2.43)) than *Oryzias latipes/Oryzias latipes* (59.84 minutes (sd +/- 2.86)). Black circle = mean Black line = 95% confidence interval. Welch two sample t-test p = 2.7E-07. N= 26 *Oryzias sakaizumii/Oryzias latipes* F1 hybrid embryos, N= 42 *Oryzias latipes/Oryzias latipes* F1 hybrid embryos.

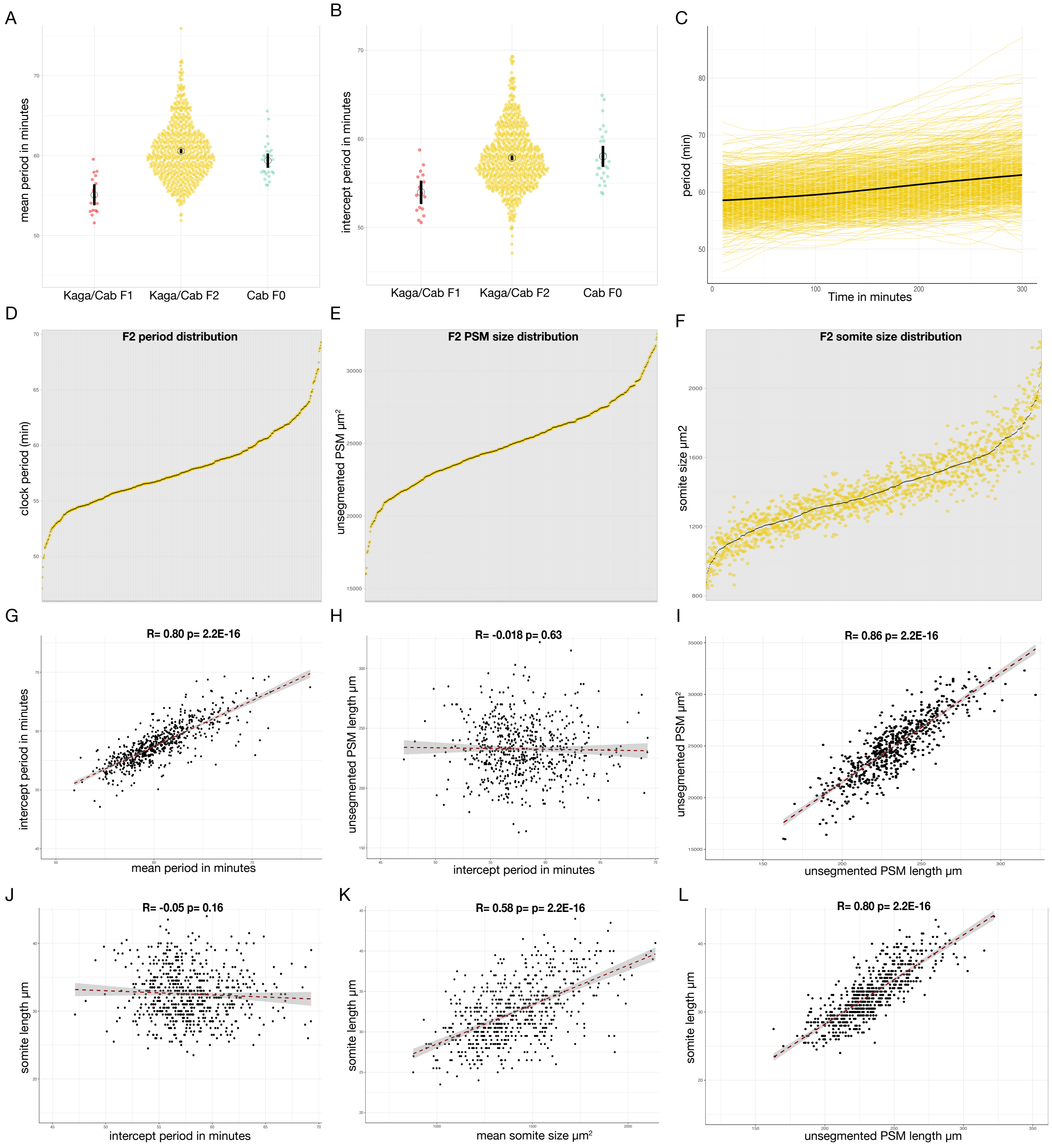

Seleit et al., Supplementary Figure 2

Appendix Figure S2. F2 Kaga/Cab *her7-venus* reveals modular nature of segmentation timing and unsegmented PSM/somite size

(A) endogenous *her7-venus* mean period measurements of F2 Kaga/Cab embryos imaged at 10-11SS (60.57 minutes (sd +/- 3.54)), compared to mean period values for F1 Kaga/Cab (55.09 minutes (sd +/- 2.39)) and F0 Cab/Cab (59.37 minutes (sd +/- 2.26)). Black circle = mean period. Black line = 95% confidence interval. Each dot is one embryo. N= 638 F2 Kaga/Cab embryos N= 16 F1 Kaga/Cab embryos N= 28 F0 Cab embryos. (B) endogenous *her7-venus* intercept period measurements of F2 Kaga/Cab embryos (57.86 minutes (sd +/- 3.43)) imaged at the 10-11SS compared to intercept period values for F1 Kaga/Cab (53.97 minutes (sd +/- 2.4)) and F0 Cab/Cab (58.03 minutes (sd +/- 3.02)). Black circle = mean period. Black line = 95% confidence interval. Each dot is one embryo. N= 638 F2 Kaga/Cab embryos N= 16 F1 Kaga/Cab embryos N= 28 F0 Cab embryos. Same panel as in Figure 3a (C) endogenous *her7-venus* period measurements over 300 minutes in F2 Kaga/Cab embryos imaged at the 10-11SS. A considerable endogenous *her7-venus* period range is obtained in the F2 cross. Black line = mean period N= 638 F2 Kaga/Cab embryos. (D) endogenous *her7-venus* clock period measurements of F2 Kaga/Cab arranged from fastest period (47.1 minutes) to slowest period (69.2 minutes). A significant period range (22.1 minutes) is obtained in the F2 cross. Each black line is an intercept period measurement from one F2 embryo. N= 638 F2 Kaga/Cab embryos. (E) Unsegmented PSM measurements of F2 Kaga/Cab arranged from smallest PSM 15986µm2 to the largest PSM 32546µm2. A significant PSM size range 16560µm2 is obtained in the F2 cross. Each black line is an unsegmented PSM measurement from one F2 embryo. N= 633 Kaga/Cab F2 (F) Somite size measurements are arranged from the smallest 875µm2 to the largest 2143µm2 . A sizable somite size range 1268µm2 is obtained in the F2 cross. Each black line is the mean somite size from one F2 embryo. Yellow dots represent individual (left and right) nascent somite size measurements. N= 631 Kaga/Cab F2. (G) Pearson's correlation between mean period (A) and intercept period (B) measurements across all F2 Kaga/Cab embryos R= 0.80 p-value= 2.2E-16. Red dotted line= linear fit, grey shaded area= 95% confidence interval N= 638 F2 Kaga/Cab embryos (H) Pearson's correlation between unsegmented PSM length and intercept period across all F2 Kaga/Cab embryos R= -0.018 p-value= 0.63. Red dotted line= linear fit, grey shaded area= 95% confidence interval. N= 623 Kaga/Cab F2. (I) Pearson's correlation between unsegmented PSM size and unsegmented PSM length across all F2 Kaga/Cab embryos R= 0.86 p-value= 2.2E-16. Red dotted line= linear fit, grey shaded area= 95% confidence interval. N= 632 Kaga/Cab F2. (J) Pearson's correlation between somite length and intercept period in minutes across all F2 Kaga/Cab embryos R= -0.05 p-value= 0.16. Red dotted line= linear fit, grey shaded area= 95% confidence interval. N= 623 Kaga/Cab F2. (K) Pearson's correlation between somite length and mean somite size across all F2 Kaga/Cab embryos R= 0.58 p-value= 2.2E-16. Red dotted line= linear fit, grey shaded area= 95% confidence interval. N= 632 Kaga/Cab F2. (L) Pearson's correlation between somite length and unsegmented PSM length across all F2 Kaga/Cab embryos R= 0.80 p-value= 2.2E-16. Red dotted line= linear fit, grey shaded area= 95% confidence interval N= 632 Kaga/Cab F2.

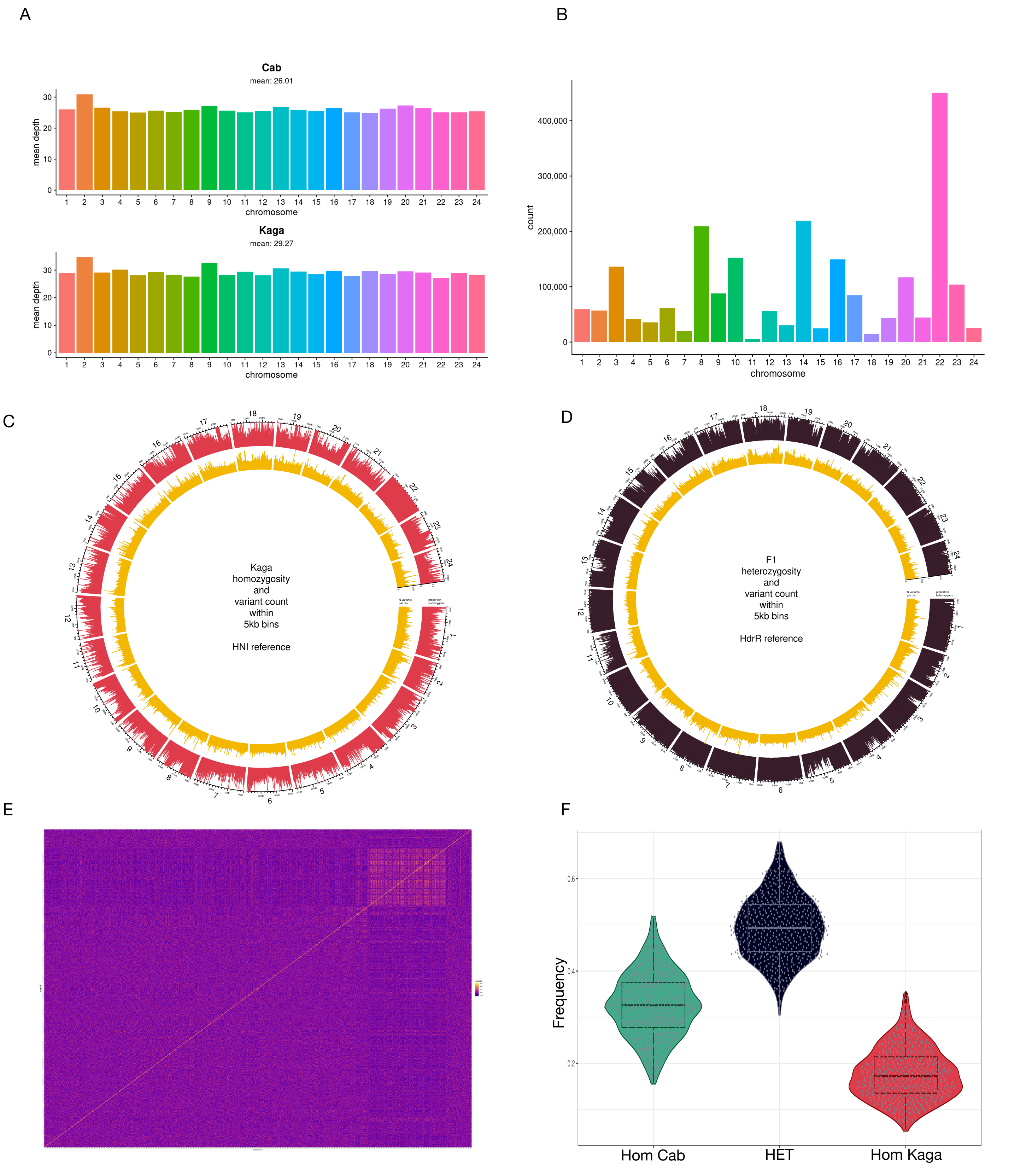

Seleit et al.,Supplementary Figure 3

Appendix Figure S3. Whole genome sequencing of F0, F1 and F2 Kaga/Cab

(A) Mean sequencing depth per chromosome for Cab and Kaga F0 strains, with genome-wide mean depth across all chromosomes shown (B) Number of SNPs per chromosome that are homozygous-divergent between F0 Cab and Kaga, and heterozygous in the F1 generation.(C) Circos plot showing the 24 chromosomes of a whole genome sequenced F0 Kaga embryo aligned against the HNI northern reference genome. Proportion of homozygous SNPs within 5kb bins in the Kaga F0 genome is shown in red and number of SNPs in each bin in yellow. The mean homozygoisty across all bins is 31% indicating a moderate level of isogenecity genome-wide (D) Circos plot showing the 24 chromosomes of a whole genome sequenced F1 hybrid Kaga/Cab embryo aligned against the HdrII reference genome. Proportion of heterozygous SNPs within 5kb bins in the F1 hybrid genome is shown in dark brown and number of SNPs in each bin in yellow. The mean heterozygosity across all bins is 67% (E) A genetic relationship matrix (GRM) constructed from the entire genome of 600 F2 samples is presented as a heatmap, with each sample represented on each axis, and lighter colours representing a higher degree of relatedness between a pair of samples. The square in the top right-hand corner is created by samples 550-648, which have distinct genotypes to the rest of the samples due to their having been bred from different F1 parents. (F)Proportions of 5-kb blocks called as either homozygous-Cab, heterozygous, or homozygous-Kaga in the F2 Kaga/Cab embryos.

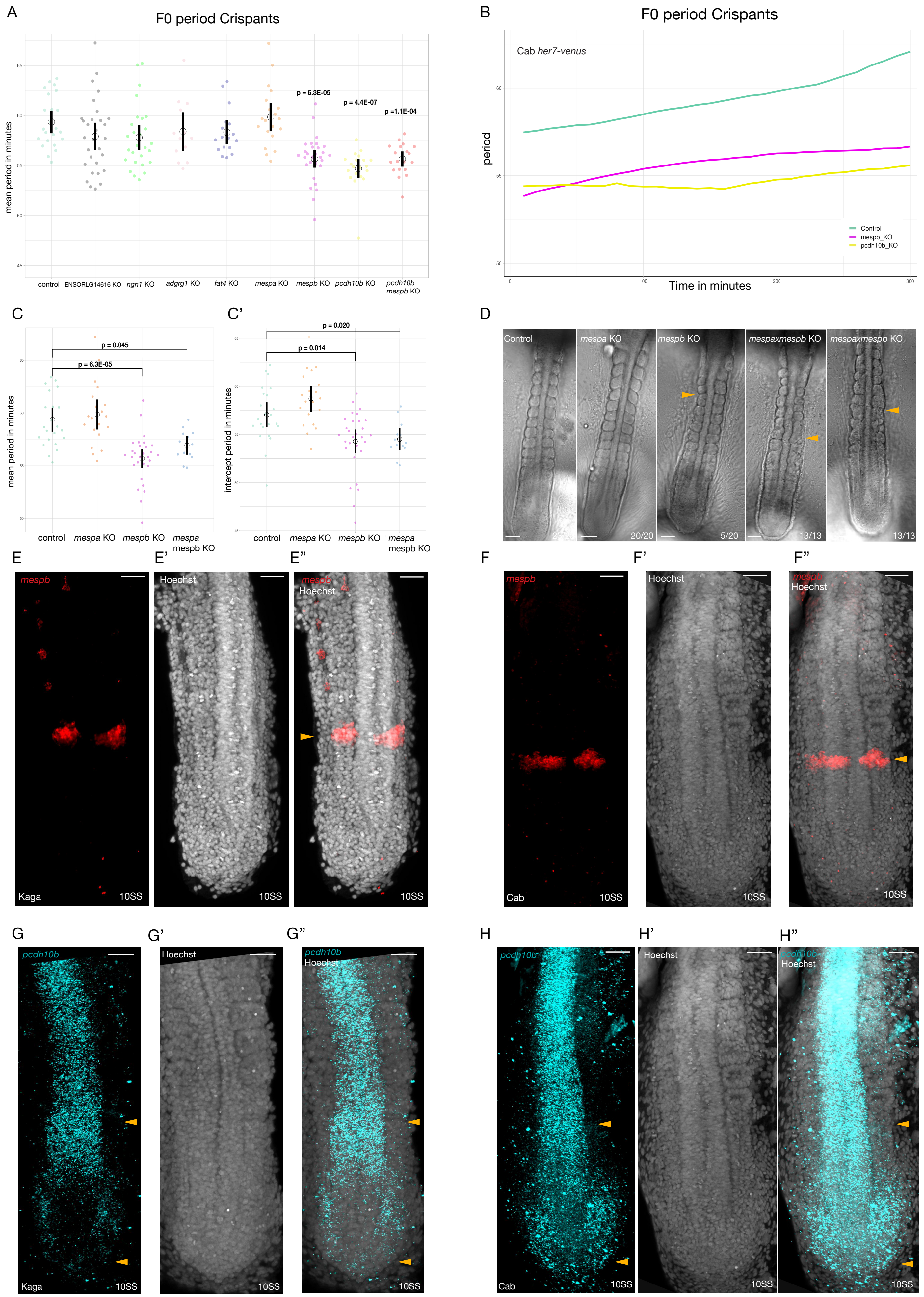

Seleit et al., Supplementary Figure 4

Appendix Figure S4. Functional validation of *dev*QTL hits using CRISPR/Cas9 KOs segmentation timing

(A) endogenous *her7-venus* mean period measurements in Control, ENSORLG14616, *ngn1, adgrg1, fat4, mespa, mespb, pcdh10b, mespb+pcdh10b* F0 Cab Crispants imaged at the 10-11SS. Kruskal-Wallis' test p= 7.8E-11. Post-Hoc Dunn's test: only *mespb* (p-adjusted=6.3E-05), *pcdh10b* (p-adjusted =4.4E-07), *mespb+pcdh10b* (p-adjusted=1.1E-04) show a significant difference in mean period as compared to control injected embryos. N= 23 control injected Cab *her7-venus* embryos, N= 30 ENSORLG14616, N= 27 *ngn1*, N= 13 a*dgrg1*, N= 17 *fat4*, N= 20 *mespa*, N= 29 *mespb*, N= 20 *pcdh10b*, N= 19 *pcdh10b+mespb* CRISPR/Cas9 injected into Cab *her7-venus*. (B) endogenous *her7-venus* posterior period measurements over 300 minutes in 10-11SS Cab F0 CRISPR/Cas9 knock-outs on candidate genes from the *dev*QTL. *mespb* (magenta) and *pcdh10b* (yellow) F0 Crispants have a faster segmentation period than Control Cas9 mRNA injected *her7-venus* Cab embryos (green). Period lines= mean values across samples. N= 23 control injected Cab *her7-venus* embryos. N= 29 *mespb* CRISPR/Cas9 injected into Cab *her7-venus*. N= 20 *pcdh10b* CRISPR/Cas9 injected into Cab *her7-venus*. (C) endogenous *her7-venus* mean period measurements in Control, *mespa*, *mespb* and *mespa+mespb* F0 Cab Crispants imaged at the 10-11SS. *mespb* and *mespa+mespb* Crispants show a faster mean *her7-venus* period (55.67 minutes (sd +/- 2.3)) and (56.92 minutes (sd +/- 1.41)) respectively than Cab control Cas9 mRNA injected embryos (59.35 minutes (sd +/- 2.56)) and *mespa* Crispants (59.85 minutes (sd +/- 2.96)). Kruskal-Wallis' test p= 7.8E-11. Post-Hoc Dunn's test: only *mespb* (p-adjusted=6.3E-05), *mespa+mespb* (p-adjusted=0.045) show a significant difference in mean period as compared to control injected embryos. N= 23 control injected Cab *her7-venus* embryos, N= 29 *mespb*, N= 20 *mespa*, N= 13 *mespa+mespb* CRISPR/Cas9 injected into Cab *her7-venus*. (C') endogenous *her7-venus* intercept period measurements in Control, *mespa, mespb* and *mespa+mespb* F0 Cab Crispants imaged at the 10-11SS. *mespb* and *mespa+mespb* Crispants show a faster intercept *her7-venus* period (54.28 minutes (sd +/- 3.2)) and (54.51 minutes (sd +/- 1.8)) respectively than Cab control Cas9 mRNA injected embryos (57,04 minutes (sd +/- 2.9)) and *mespa* Crispants (58.69 minutes (sd +/- 2.81)) Kruskal-Wallis' test p= 8.9E-08. Post-Hoc Dunn's test: only *mespb* (p-adjusted=0.014), *mespa+mespb* (p-adjusted=0.020) show a significant difference in intercept period as compared to control injected embryos. N= 23 control injected Cab *her7-venus* embryos, N= 20 *mespa*, N= 29 *mespb*, N= 13 *mespa+mespb* CRISPR/Cas9 injected into Cab *her7-venus*.(D) Brightfield imaging of tails of Control, *mespa, mespb* and *mespa+mespb* F0 Cab Crispants imaged at the 10-11SS. *mespa* Crispants (20/20) show no morphological somite phenotype, while a minority *mespb* Crispants (5/20) show mild defects in one or more somite boundaries and size (yellow arrowheads). All *mespa+mespb* (13/13) double Crispants show severe defects in somite boundary formation and somite size (yellow arrowheads). Scale bar= 50µm. (E-E'') HCR on *mespb* performed on Kaga embryos at the 10SS. (E) *mespb* is expressed as one stripe at the S-1 somite position (E') Hoechst labels all nuclei in the tail (E'') overlay of *mespb* and Hoechst from (E-E'), yellow arrowhead highlights position of the mespb stripe in relation to the forming somites in the tail. N= 8 embryos, Scale bar= 30µm. (F-F'') HCR on *mespb* performed on Cab embryos at the 10SS. (F) *mespb* is expressed as one stripe at the S-1 somite position (F') Hoechst labels all nuclei in the tail (F'') overlay of *mespb* and Hoechst from (F-F'), yellow arrowhead highlights position of the *mespb* stripe in relation to the forming somites in the tail. N= 12 embryos, Scale bar= 40µm. (G-G'') HCR on *pcdh10b* performed on Kaga embryos at the 10SS. (G) *pcdh10b* is expressed in the neural tube in addition to the unsegmented PSM. Anterior and posterior PSM positions are highlighted by yellow arrowheads (G') Hoechst labels all nuclei in the tail (G'') overlay of *pcdh10b* and Hoechst from (G-G'), yellow arrowhead highlights position of anterior and posterior PSM. N= 9 embryos, Scale bar= 30µm. (H-H'') HCR on *pcdh10b* performed on Cab embryos at the 10SS. (H) *pcdh10b* is expressed in the neural tube in addition to the unsegmented PSM. Anterior and posterior PSM positions are highlighted by yellow arrowheads, higher expression levels in the PSM domain and an extended expression in the neural tube are observed as compared to Kaga embryos (H') Hoechst labels all nuclei in the tail. Same embryo shown in (F') (H'') overlay of *pcdh10b* and Hoechst from (G-G'), yellow arrowhead highlights position of anterior and posterior PSM. N= 8 embryos, Scale bar= 40µm.

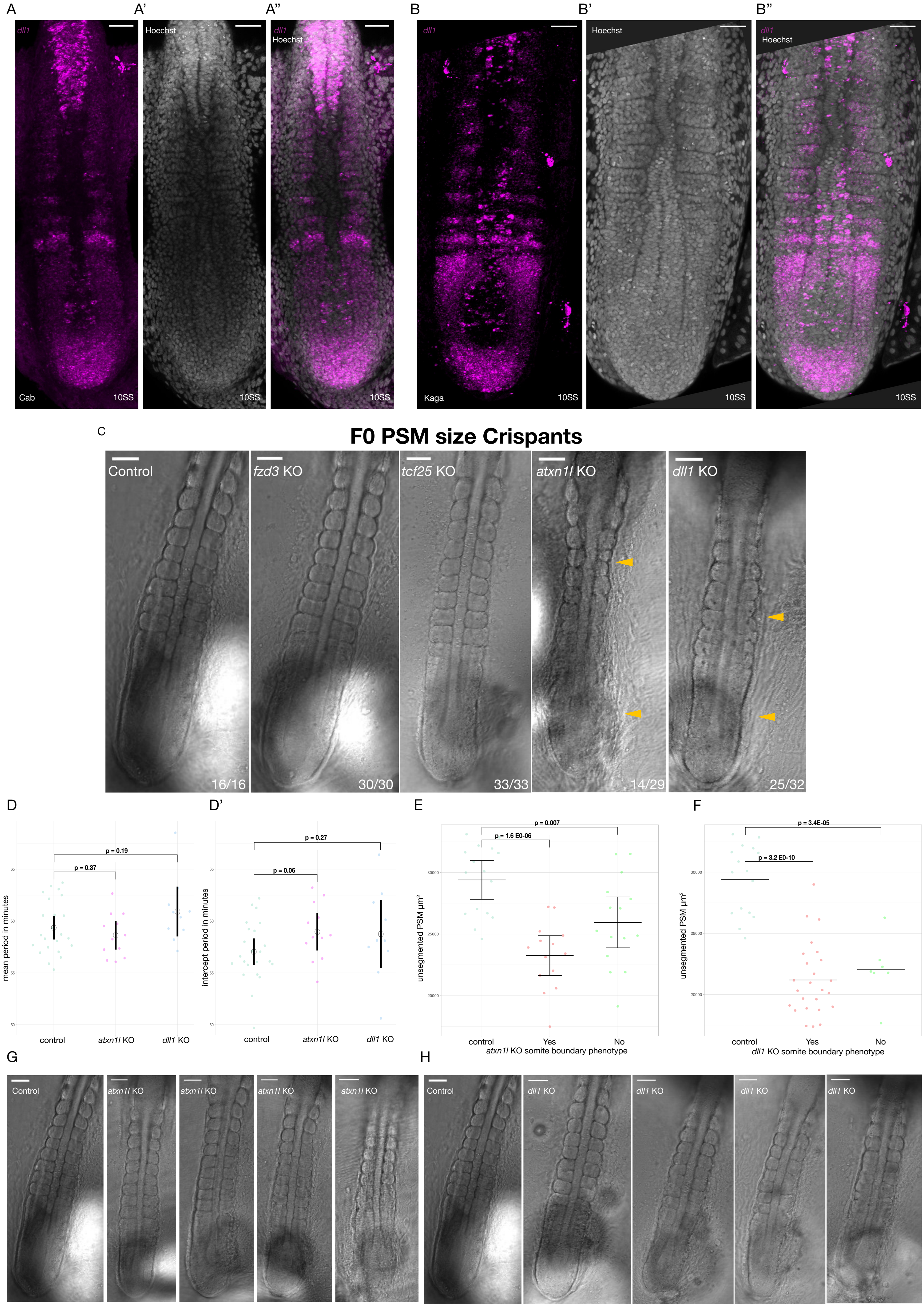

Seleit et al., Supplementary Figure 5

Appendix Figure S5. Functional validation of *dev*QTLs on unsegmented PSM size using F0 Crispants and HCR

(A-A'') HCR on *dll1* performed on Cab embryos at the 10SS. (A) *dll1* is expressed throughout the unsegmented PSM (posterior and anterior), in addition to striped expression in formed somites (A') Hoechst labels all nuclei in the tail (A'') overlay of *dll1* and Hoechst from (A-A'). N= 6 embryos, Scale bar= 40µm. (b-b'') HCR on *dll1* performed on Kaga embryos at the 10SS. (B) *dll1* is expressed throughout the unsegmented PSM (posterior and anterior), in addition to striped expression in formed somites (B') Hoechst labels all nuclei in the tail (b'') overlay of *dll1* and Hoechst from (b-b'). N= 7 embryos, Scale bar= 40µm. (C) unsegmented PSM size Cab F0 CRISPR/Cas9 knock-outs performed on candidate genes from the *dev*QTL mapping results, brightfield imaging was carried out on Control, *fzd3*, *tcf25, atxn1l* and *dll1* F0 Cab Crispants imaged at the 10-11 somite stage. Both *atxn1l* and *dll1* Crispants showed smaller unsegmented PSM sizes than control injected embryos (yellow arrow-heads), in addition both *atxn1l* (14/29) and *dll1* (25/32) Crispants showed morphological somite size and boundary abnormalities (yellow arrow-heads). N= 16 control injected embryos,N = 30 *fzd3* Crispants N= 33 *tcf25* Crispants N= 29 *atxn1l* Crispants N= 32 *dll1* Crispants. Scale bar= 50µm.(D) endogenous *her7-venus* mean period measurements in Control, *atxn1l* and *dll1* F0 Cab Crispants imaged at the 10-11 somite stage. Both Crispants show a similar mean *her7-venus* period (58.63 minutes (sd +/- 2.08)) and (60.92 minutes (sd +/- 3.18)) respectively to Cab control Cas9 mRNA injected embryos (59.35 minutes (sd +/- 2.56)). Welch two sample t-test p = 0,37 for *atxn1l* Crispants and p = 0,19 for *dll1* Crispants. N= 23 control injected Cab *her7-venus* embryos. N= 12 *atxn1l,* N= 10 *dll1* CRISPR/Cas9 F0 injected into Cab *her7-venus.* (d') endogenous *her7-venus* intercept period measurements in Control, *atxn1l* and *dll1* F0 Cab Crispants imaged at the 10-11 somite stage. Both Crispants show a similar intercept *her7-venus* period (58.97 minutes (sd +/- 2.73)) and (58.75 minutes (sd +/- 4.34)) respectively to Cab control Cas9 mRNA injected embryos (57.04 minutes (sd +/- 2.90)). Welch two sample t-test p = 0.064 for *atxn1l* Crispants and p = 0.27 for *dll1* Crispants. N= 23 control injected Cab *her7-venus* embryos. N= 12 *atxn1l,* N= 10 *dll1* CRISPR/Cas9 F0 injected into Cab *her7-venus.* (E) unsegmented PSM size measurements on 10-11 somite stage Cab F0 CRISPR/Cas9 knock-outs separated by the presence or absence of a somite morphology phenotype in *atxn1l* Crispants. *atxn1l* Cripsants with no somite phenotype still showed significantly smaller unsegmented PSM size (25947µm^2 (sd +/- 3605)) when compared to Cab control Cas9 mRNA injected embryos (29392µm^2 (sd +/- 2851)). *atxn1l* Cripsants with a somite phenotype also showed significantly smaller unsegmented PSM size (23251µm^2 (sd +/- 2689)) compared to Control injected samples. Welch two sample t-test p = 7.1E-03 for *atxn1l* Crispants with no somite phenotype and p = 1,5E-06 for *atxn1l* Crispants with a somite phenotype. N = 16 control injected embryos N= 15 *atxn1l* Crispants with no somite phenotype N= 14 *atxn1l* Crispants with visible somite phenotype (F) unsegmented PSM size measurements on 10-11SS Cab F0 CRISPR/Cas9 knock-outs separated by the presence or absence of a somite phenotype in *dll1* Crispants. *dll1* Cripsants with no somite phenotype (minority of samples) showed significantly smaller unsegmented PSM size (22064µm^2 (sd +/- 2513)) when compared to Cab control Cas9 mRNA injected embryos (29392µm^2 (sd +/- 2851)). *dll1* Cripsants with a somite phenotype showed significantly smaller unsegmented PSM size (21191µm^2 (sd +/- 2977)) compared to Control injected samples. Welch two sample t-test p = 3.4E-05 for *dll1* Crispants with no somite phenotype and p = 3.19E-10 for *dll1* Crispants with a somite phenotype. N = 16 control injected embryos N= 7 *dll1* Crispants with no somite phenotype N= 25 *dll1* Crispants with visible somite phenotype (G) Brightfield imaging on control injected embryos, same embryo shown in (C) and *atxn1l* F0 Cab Crispants imaged at the 10-11SS showing no somite phenotype. Example of 3 *atxn1l* Crispants with smaller unsegmented PSM sizes and no visible somite phenotypes compared to control injected embryos. N= 16 control injected embryos, N = 15 *atxn1l* Crispants with no somite morphology phenotype. Scale bar= 50µm. (H) Brightfield imaging on control injected embryo same embryo shown in (C, G) and *dll1* F0 Cab Crispants imaged at the 10-11SS showing no visible somite phenotype. Example of 3 *dll1* Crispants with smaller unsegmented PSM sizes and no somite morphology phenotype compared to control injected embryos (same embryo as in H). N= 16 control injected embryos, N= 7 *dll1* Crispants with no somite morphology phenotype. Scale bar= 50µm.
