# Supplementary material for: Modular control of vertebrate axis segmentation in time and space
Source: EMBO J. 2024 Aug 9;43(18):4068–91. doi: 10.1038/s44318-024-00186-2 (PMC11405765; doi:10.1038/s44318-024-00186-2)
Supplement: Supplementary file 13 — Expanded View Figures [file 44318_2024_186_MOESM13_ESM.pdf]

Expanded View Figures

Figure EV1. Scaling of embryonic segmentation timing and size in the *Oryzias* genus.

(**A**) Pearson's correlation of average trait values for adult mass and adult length (11 months) in *O. minutillus* (pink), *O. hubbsi* (blue), *O. mekongensis* (orange), *O. sakaizumii* (light blue) and *O. latipes* (magenta) shows a positive correlation $R = 0.99$ P value $= 1.0E-03$. Black dotted line= linear fit on average trait values, vertical and horizontal lines on both axes represent standard deviation (SD). $N = 10$ *O. minutillus*, $N = 10$ *O. minutillus*, $N = 12$ *O. hubbsi*, $N = 10$ *O. mekongensis*, $N = 13$ *O. sakaizumii*, $N = 15$ *O. latipes* for adult length and $N = 6$ each *O. hubbsi*, *O. mekongensis, O. sakaizumii*, *O. latipes* for adult mass. (**B**) Pearson's correlation of average trait values for adult mass and embryonic segmentation rate in *O. minutillus* (pink), *O. hubbsi* (blue), *O. mekongensis* (orange), *O. sakaizumii* (light blue) and *O. latipes* (magenta) shows a positive correlation $R = 0.94$ P value $= 1.9E-02$. Black dotted line= linear fit on average trait values, vertical and horizontal lines on both axes represent standard deviation (SD). $N = 10$ *O. minutillus*, $N = 10$ *O. minutillus*, $N = 12$ *O. hubbsi*, $N = 10$ *O. mekongensis*, $N = 13$ *O. sakaizumii*, $N = 15$ *O. latipes* for adult length and $N = 11$ *O. minutillus*, $N = 10$ *O. hubbsi*, $N = 10$ *O. mekongensis*, $N = 20$ *O. sakaizumii*, $N = 30$ *O. latipes* for segmentation rate. (**C**) Bright-field image of unsegmented presomitic mesoderm (PSM) and nascent somites at the 10-11 somite stage (SS) Cab F0 medaka embryo. Yellow dotted line delineates the unsegmented PSM area while white dotted lines delineate the area of nascent somites. Scale bar= 50 µm. (**D**) Schematic map of mainland Japan (showing Honshu, Shikoku and parts of Kyushu) highlighting the sites of the original medaka populations. The northern *Oryzias sakaizumii*: HNI (blue) and Kaga (red) diverged from the southern *Oryzias latipes*: Cab (turquoise), HdrII (magenta) and Ho5 (yellow) 18 million years ago. (**E**) Pearson's correlation of average trait values for nascent somite size and somite length measured at 10-11 somite stage in *O. minutillus* (pink), *O. hubbsi* (blue), *O. mekongensis* (orange), *O. sakaizumii* Kaga (red), HNI (light blue) and *O. latipes* Cab (green), HdrII (magenta), Ho5 (yellow) embryos shows a positive correlation $R = 0.95$ P value $= 1.5E-02$. Black dotted line= linear fit on average trait values, vertical and horizontal lines on both axes represent standard deviation (SD). Individual data points are shown for each population $N = 11$ *O. minutillus*, $N = 13$ *O. hubbsi*, $N = 18$ *O. mekongensis*, $N = 19$ Cab, $N = 10$ HdrII, $N = 7$ Ho5, $N = 20$ Kaga, $N = 10$ HNI. (**F**) Pearson's correlation of average trait values for unsegmented PSM length and unsegmented PSM area measured at 10-11 somite stage in *O. minutillus* (pink), *O. hubbsi* (blue), *O. mekongensis* (orange), *O. sakaizumii* Kaga (red), HNI (light blue) and *O. latipes* Cab (green), HdrII (magenta), Ho5 (yellow) embryos shows a positive correlation $R = 0.99$ P value $= 3.2E-04$. Black dotted line= linear fit on average trait values, vertical and horizontal lines on both axes represent standard deviation (SD). Individual data points are shown for each population $N = 11$ *O. minutillus*, $N = 13$ *O.hubbsi*, $N = 18$ *O. mekongensis*, $N = 19$ Cab, $N = 10$ HdrII, $N = 7$ Ho5, $N = 20$ Kaga, $N = 10$ HNI. (**G**) Pearson's correlation of average trait values for unsegmented PSM length and somite length measured at 10-11 somite stage in *O. minutillus* (pink), *O. hubbsi* (blue), *O. mekongensis* (orange), *O. sakaizumii* Kaga (red), HNI (light blue) and *O. latipes* Cab (green), HdrII (magenta), Ho5 (yellow) embryos shows a positive correlation $R = 0.96$ P value $= 9.1E-03$. Black dotted line= linear fit on average trait values, vertical and horizontal lines on both axes represent standard deviation (SD). Individual data points are shown for each population $N = 11$ *O. minutillus*, $N = 13$ *O. hubbsi*, $N = 18$ *O. mekongensis*, $N = 19$ Cab, $N = 10$ HdrII, $N = 7$ Ho5, $N = 20$ Kaga, $N = 10$ HNI. (**H**) Axis segmentation rate from brightfield time-lapse imaging of Kaga and Cab F0 embryos measured at the 10-11 somite stage calculated from the time it takes to form 5 consecutive pairs of somites. Kaga embryos have a faster segmentation period (52.88 min (SD $+/-$ 2.42)) than Cab (60.84 min (SD $+/-$ 2.52)). Welch two sample *t* test $P = 4.3E-12$. Black circle = mean Black line = 95% confidence interval. $N = 20$ Kaga embryos, $N = 19$ Cab embryos. (**I**) Ex-vivo explant axis segmentation rate in bright-field imaging of Kaga and Cab F0 embryos at the 15-16 somite stage calculated from the time it takes to form 5-6 consecutive pairs of somites. Kaga embryos have a faster ex-vivo segmentation period (63.47 min (SD $+/-$ 4.2)) than Cab (73.75 min (SD $+/-$ 5.9)). Welch two sample *t* test $P = 2.8E-05$. Black circle = mean Black line = 95% confidence interval. $N = 13$ Kaga embryos, $N = 14$ Cab embryos.

© The Author(s)

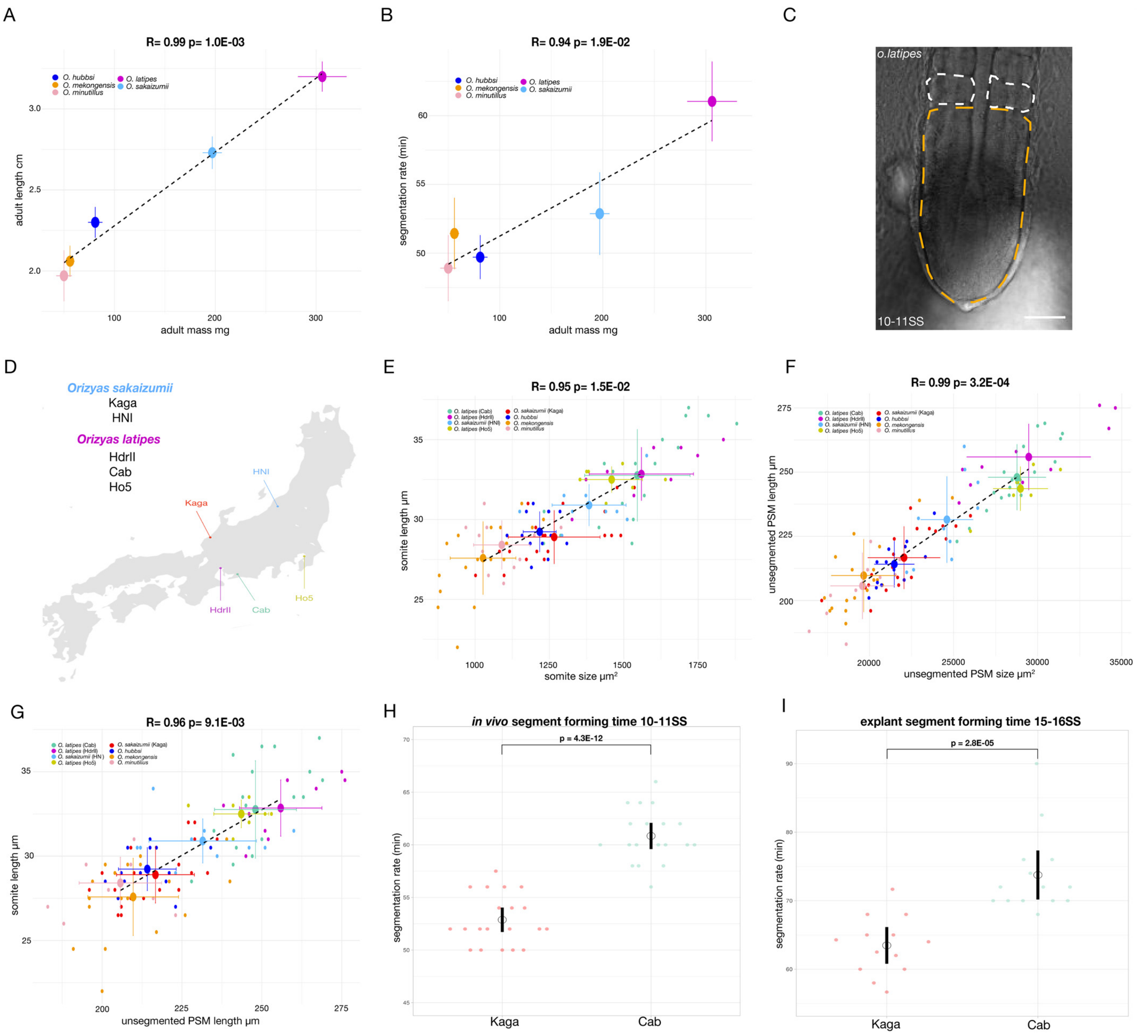
A
R= 0.99 p= 1.0E-03
O. hubbsi
O. mekongensis
O. minutillus
O. latipes
O. sakaizumii
adult length cm
adult mass mg
B
R= 0.94 p= 1.9E-02
segmentation rate (min)
adult mass mg
C
o.latipes
10-11SS
D
Orizyas sakaizumii
Kaga
HNI
Orizyas latipes
HdrII
Cab
Ho5
E
R= 0.95 p= 1.5E-02
somite length µm
somite size µm²
F
R= 0.99 p= 3.2E-04
unsegmented PSM length µm
unsegmented PSM size µm²
G
R= 0.96 p= 9.1E-03
somite length µm
unsegmented PSM length µm
H
in vivo segment forming time 10-11SS
p = 4.3E-12
segmentation rate (min)
Kaga
Cab
I
explant segment forming time 15-16SS
p = 2.8E-05
segmentation rate (min)
Kaga
Cab

© The Author(s)

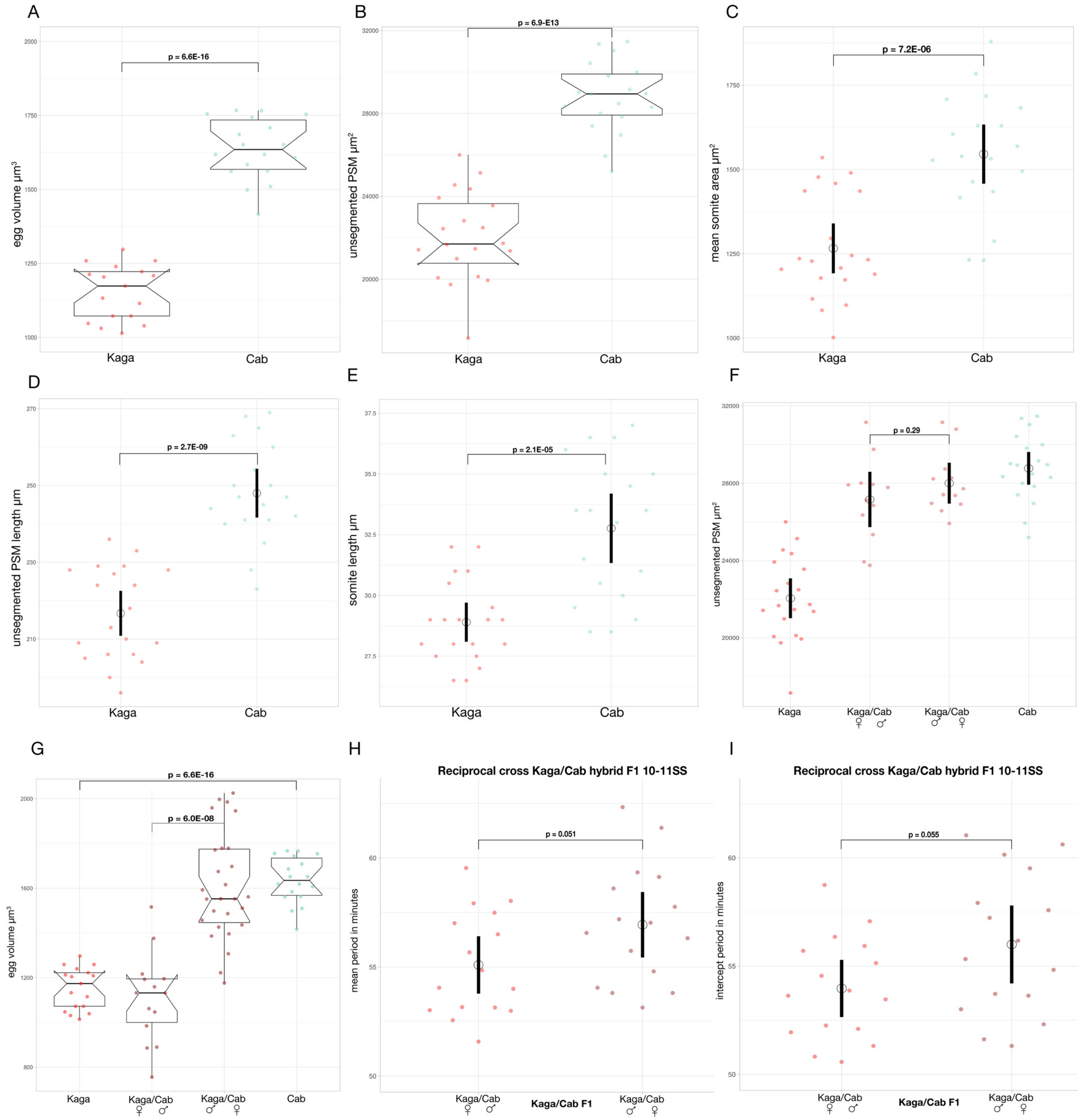
A
p = 6.6E-16
egg volume µm³
Kaga
Cab
B
p = 6.9-E13
unsegmented PSM µm²
Kaga
Cab
C
p = 7.2E-06
mean somite area µm²
Kaga
Cab
D
p = 2.7E-09
unsegmented PSM length µm
Kaga
Cab
E
p = 2.1E-05
somite length µm
Kaga
Cab
F
p = 0.29
unsegmented PSM µm²
Kaga
Kaga/Cab ♀ ♂
Kaga/Cab ♂ ♀
Cab
G
p = 6.6E-16
p = 6.0E-08
egg volume µm³
Kaga
Kaga/Cab ♀ ♂
Kaga/Cab ♂ ♀
Cab
H
Reciprocal cross Kaga/Cab hybrid F1 10-11SS
p = 0.051
mean period in minutes
Kaga/Cab ♀ ♂
Kaga/Cab F1
Kaga/Cab ♂ ♀
I
Reciprocal cross Kaga/Cab hybrid F1 10-11SS
p = 0.055
intercept period in minutes
Kaga/Cab ♀ ♂
Kaga/Cab F1
Kaga/Cab ♂ ♀

◀ **Figure EV2. Kaga and Cab F0 and reciprocal hybrid F1 segmentation timing and size measurements.**

(**A**) F0 Kaga have smaller fertilised egg volume (1152 µm^3 (SD +/− 93,0)) than Cab (1636 µm^3 (SD +/− 103)). Welch two sample *t* test *P* = 6.6E-16. *N* = 17 Kaga *N* = 18 Cab eggs. (**B**) Area of unsegmented PSM at the 10-11 somite stage of Kaga and Cab F0 embryos. Kaga embryos have smaller unsegmented PSM (22,048 µm^2 (SD +/− 2145)) compared to Cab (28,770 µm^2 (SD +/− 1709)). Welch two sample *t* test *P* = 6.9E-13. *N* = 20 Kaga *N* = 19 Cab embryos. (**C**) somite area of nascent pair of somites in Kaga and Cab F0 embryos at the 10-11 somite stage. Kaga embryos have smaller somites (1265 µm^2 (SD +/− 154)) than Cab (1545 µm^2 (SD +/− 177)) embryos. Black circle = mean Black line = 95% confidence interval. Welch two sample *t* test *P* = 7.20E-06. *N* = 20 Kaga embryos, *N* = 19 Cab embryos. (**D**) Length of unsegmented PSM at the 10SS of Kaga and Cab F0 embryos. Kaga embryos have shorter unsegmented PSM (216.7 µm (SD +/− 12.19)) compared to Cab (248 µm (SD +/− 12.83)). Black circle = mean Black line = 95% confidence interval. Welch two sample *t* test *P* = 2.7E-09. *N* = 20 Kag*a*, *N* = 19 Cab embryos. (**E**) Length of nascent somite measured at the 10SS of Kaga and Cab F0 embryos. Kaga embryos have shorter somites (28.9 µm (SD +/− 1.67)) compared to Cab (32.76µm (SD +/− 2.88)). Black circle = mean Black line = 95% confidence interval. Welch two sample *t* test *P* = 2.1E-05. *N* = 20 Kaga *N* = 19 Cab embryos. (**F**) Area of unsegmented PSM µm^2 at the 10-11SS of Kaga/Cab F1 reciprocal cross compared to the paternal F0 Kaga and Cab populations. Kaga/Cab F1 embryos coming from Kaga females crossed to Cab male embryos have unsegmented PSM size of (2716 µm^2 (SD +/− 1592)) compared Kaga/Cab F1 embryos coming from Kaga males crossed to Cab females (28,004 µm^2 (SD +/− 2154)). While the Kaga F0 population embryos have an unsegmented PSM size of (22,048 µm^2 (SD +/− 2145)) compared to Cab (28,770 µm^2 (SD +/− 1709)). Black circle = mean Black line = 95% confidence interval. Welch two sample *t* test *P* = 0.29. *N* = 20 Kaga, *N* = 19 Cab embryos. *N* = 12 Kaga/Cab F1 embryos coming from a cross of Kaga females to Cab males, *N* = 12 Kaga/Cab F1 embryos coming from a cross of Kaga males to Cab females. (**G**) Fertilised egg volume of Kaga/Cab F1 reciprocal cross shows a significant maternal effect. Kaga/Cab F1 embryos coming from Kaga females crossed to Cab male embryos have fertilised egg volume of (1110 µm^3 (SD +/− 198)) compared Kaga/Cab F1 embryos coming from Kaga males crossed to Cab females 1605 µm^3 (SD +/-236)). Welch two sample *t* test *P* = 6.0E0-08. *N* = 14 Kaga/Cab F1 embryos coming from a cross of Kaga females to Cab males, *N* = 27 Kaga/Cab F1 embryos coming from a cross of Kaga males to Cab females. F0 Kagas have smaller fertilised egg volume (1152 µm^3 (SD +/− 93.0)) than Cab (1636 µm^3 (SD +/− 103)). Black circle = mean Black line = 95% confidence interval. Welch two sample *t* test *P* = 6.6E-16. *N* = 17 Kaga, *N* = 18 Cab eggs. (**H**) endogenous *her7-venus* mean period measurements in hybrid F1 Kaga/Cab reciprocal cross at the 10-11SS shows non-significant difference. Kaga/Cab F1 embryos coming from Kaga females crossed to Cab males have a mean *her7-venus* period of (55.09 min (SD +/− 2.39)) while Kaga/Cab F1 embryos coming from Kaga males crossed to Cab females have a mean period of (56.94 min (SD +/− 2.73)). Black circle = mean Black line = 95% confidence interval. Welch two sample *t* test *P* = 0.051. *N* = 16 Kaga/Cab F1 embryos coming from a cross of Kaga females to Cab males, *N* = 16 Kaga/Cab F1 embryos coming from a cross of Kaga males to Cab females. (**I**) Endogenous *her7-venus* intercept period measurements in hybrid F1 Kaga/Cab reciprocal cross at the 10-11SS shows non-significant difference. Kaga/Cab F1 embryos coming from Kaga females crossed to Cab males have an intercept *her7-venus* period of (53.97 min (SD +/− 2.40)) while Kaga/Cab F1 embryos coming from Kaga males crossed to Cab females have a mean period of (56.00 min (SD +/− 3.27)). Black circle = mean Black line = 95% confidence interval. Welch two sample *t* test *P* = 0.055. *N* = 16 Kaga/Cab F1 embryos coming from a cross of Kaga females to Cab males, *N* = 16 Kaga/Cab F1 embryos coming from a cross of Kaga males to Cab females.

 © The Author(s)

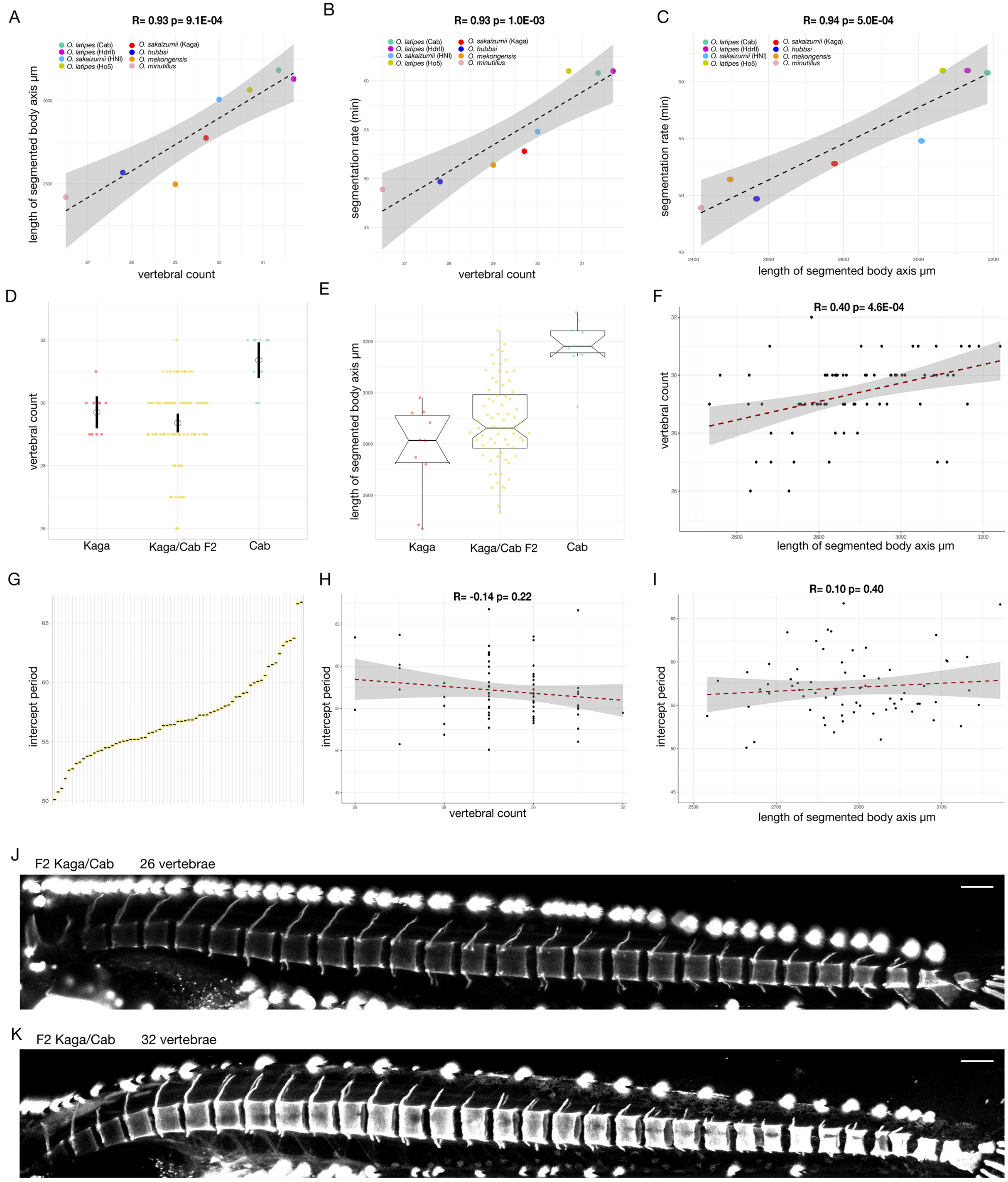
A
R= 0.93 p= 9.1E-04
O. latipes (Cab)
O. latipes (Hdrll)
O. sakaizumii (HNI)
O. latipes (Ho5)
O. sakaizumii (Kaga)
O. hubbsi
O. mekongensis
O. minutillus
length of segmented body axis μm
vertebral count
B
R= 0.93 p= 1.0E-03
segmentation rate (min)
vertebral count
C
R= 0.94 p= 5.0E-04
segmentation rate (min)
length of segmented body axis μm
D
vertebral count
Kaga
Kaga/Cab F2
Cab
E
length of segmented body axis μm
Kaga
Kaga/Cab F2
Cab
F
R= 0.40 p= 4.6E-04
vertebral count
length of segmented body axis μm
G
intercept period
H
R= -0.14 p= 0.22
intercept period
vertebral count
I
R= 0.10 p= 0.40
intercept period
length of segmented body axis μm
J
F2 Kaga/Cab
26 vertebrae
K
F2 Kaga/Cab
32 vertebrae

© The Author(s)

Figure EV3. Larval size and vertebral count do not correlate with segmentation timing in a subset of F2 embryos.

(**A**) Pearson's correlation of average trait values for vertebral count and length of segmented body axis in stage 42 *O. minutillus* (pink), *O. hubbsi* (blue), *O. mekongensis* (orange), HNI (light blue), Kaga (red) and Cab (turquoise), HdrII (magenta), Ho5 (yellow) larvae shows a positive correlation $R = 0.93$ P value = 9.1E-04. Black dotted line= linear fit on average trait values, shaded area = 95% confidence interval. $N = 10$ *O. hubbsi*, *O. mekongensis*, Kaga, HNI, Ho5, Cab, HdrII larvae $N = 11$ *O. minutillus*. (**B**) Pearson's correlation of average trait values for vertebral count and segmentation rate in minutes in *O. minutillus* (pink), *O. hubbsi* (blue), *O. mekongensis* (orange), HNI (light blue), Kaga (red) and Cab (aquamarine), HdrII (magenta), Ho5 (yellow) larvae shows a positive correlation $R = 0.93$ P value = 1.0E-03. Black dotted line= linear fit on average trait values, shaded area = 95% confidence interval. $N = 10$ *O. hubbsi*, *O. mekongensis*, Kaga, HNI, Ho5, Cab, HdrII, $N = 11$ *O. minutillus*. (**C**) Pearson's correlation of average trait values for length of segmented body axis in stage 42 larvae and segmentation rate in minutes in *O. minutillus* (pink), *O. hubbsi* (blue), *O. mekongensis* (orange), HNI (light blue), Kaga (red) and Cab (aquamarine), HdrII (magenta), Ho5 (yellow) larvae shows a positive correlation $R = 0.94$ P value = 5.0E-04. Black dotted line= linear fit on average trait values, shaded area = 95% confidence interval. $N = 10$ *O. hubbsi*, *O. mekongensis*, Kaga, HNI, Ho5, Cab, HdrII, $N = 11$ *O. minutillus*. (**D**) Vertebral count in F2 stage 42 larvae compared to Cab and Kaga F0 populations. Black circle = mean values. Black line = 95% confidence interval. Each dot is one embryo. $N = 72$ F2 Kaga/Cab embryos $N = 10$ F1 Kaga embryos $N = 11$ F0 Cab embryos. (**E**) Length of segmented body axis in stage 42 F2 larvae compared to Cab and Kaga F0 populations. Black circle = mean values. Black line = 95% confidence interval. Each dot is one embryo. $N = 72$ F2 Kaga/Cab embryos $N = 10$ F1 Kaga embryos $N = 10$ F0 Cab embryos. (**F**) Pearson's correlation between length of segmented body axis and vertebral count across a subset of F2 Kaga/Cab embryos $R = = 0.4$ P value = 4.6E-04. Red dotted line= linear fit, grey shaded area= 95% confidence interval. $N = 72$ Kaga/Cab F2. (**G**) Endogenous her7-venus intercept clock period measurements of F2 Kaga/Cab arranged from fastest to slowest. Each black line is an intercept period measurement from one F2 embryo. $N = 70$ F2 Kaga/Cab embryos. (**H**) Pearson's correlation between vertebral count and intercept clock period across a subset of F2 Kaga/Cab embryos $R = = -0.14$ P value = 0.22. Red dotted line= linear fit, grey shaded area= 95% confidence interval. $N = 70$ Kaga/Cab F2. (**I**) Pearson's correlation between length of segmented body axis and intercept clock period across a subset of F2 Kaga/Cab embryos $R = = 0.10$ P value = 0.40. Red dotted line= linear fit, grey shaded area= 95% confidence interval. $N = 70$ Kaga/Cab F2. (**J**, **K**) ALC bone staining on stage 42 F2 larvae showing the upper and lower bound (26–32) of vertebral counts obtained in the subset of F2 larvae analysed.

© The Author(s)

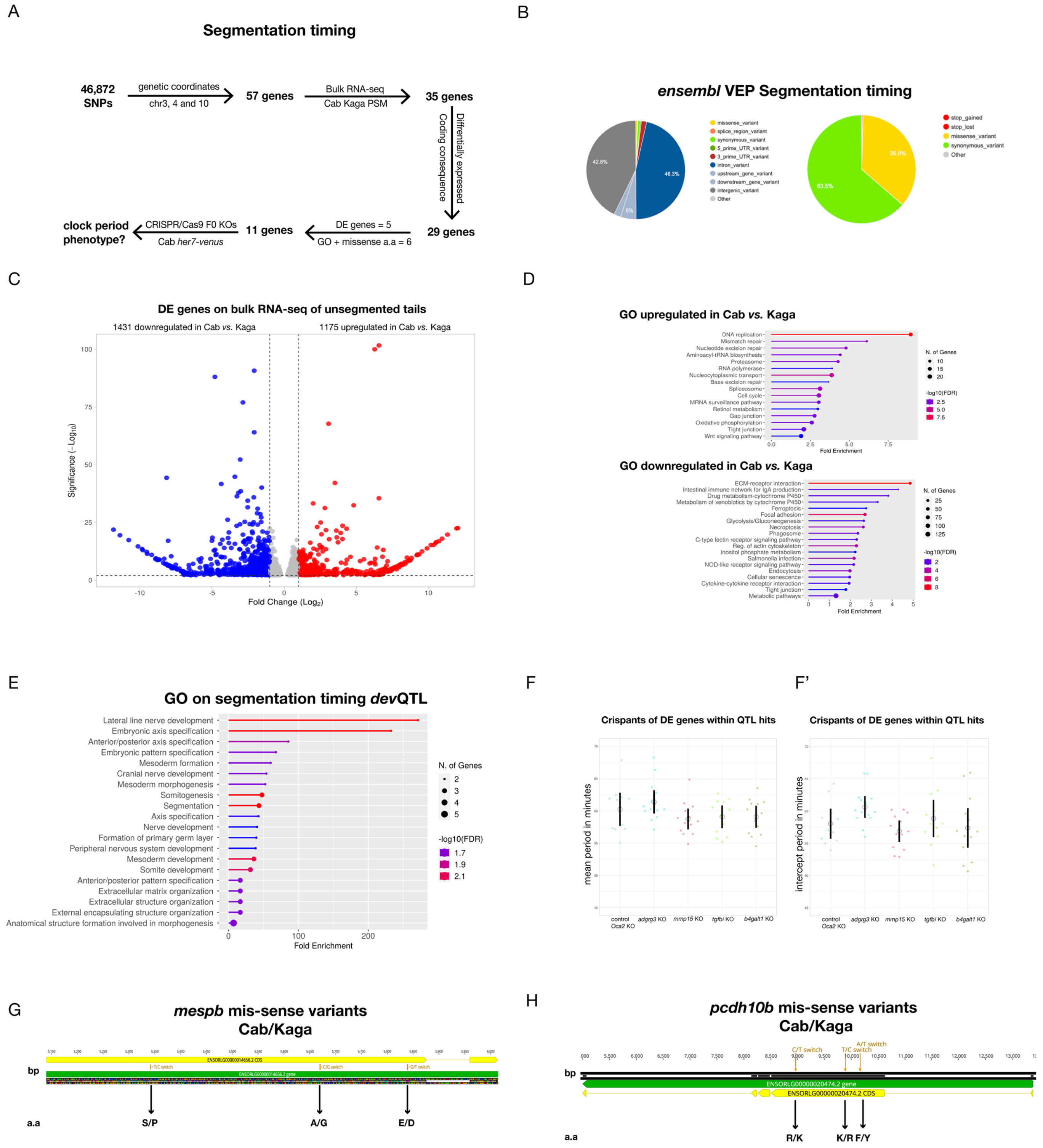

A
Segmentation timing
46,872 SNPs
genetic coordinates
chr3, 4 and 10
57 genes
Bulk RNA-seq
Cab Kaga PSM
35 genes
Differentially expressed
Coding consequence
29 genes
DE genes = 5
GO + missense a.a = 6
11 genes
CRISPR/Cas9 F0 KOs
Cab her7-venus
clock period phenotype?
B
ensembl VEP Segmentation timing
missense_variant
splice_region_variant
synonymous_variant
5_prime_UTR_variant
3_prime_UTR_variant
intron_variant
upstream_gene_variant
downstream_gene_variant
intergenic_variant
Other
42.8%
46.3%
5%
stop_gained
stop_lost
missense_variant
synonymous_variant
Other
35.9%
63.5%
C
DE genes on bulk RNA-seq of unsegmented tails
1431 downregulated in Cab vs. Kaga
1175 upregulated in Cab vs. Kaga
Significance (−Log10)
Fold Change (Log2)
D
GO upregulated in Cab vs. Kaga
DNA replication
Mismatch repair
Nucleotide excision repair
Aminoacyl-tRNA biosynthesis
Proteasome
RNA polymerase
Nucleocytoplasmic transport
Base excision repair
Spliceosome
Cell cycle
MRNA surveillance pathway
Retinol metabolism
Gap junction
Oxidative phosphorylation
Tight junction
Wnt signaling pathway
Fold Enrichment
N. of Genes
-log10(FDR)
GO downregulated in Cab vs. Kaga
ECM-receptor interaction
Intestinal immune network for IgA production
Drug metabolism-cytochrome P450
Metabolism of xenobiotics by cytochrome P450
Ferroptosis
Focal adhesion
Glycolysis/Gluconeogenesis
Necroptosis
Phagosome
C-type lectin receptor signaling pathway
Reg. of actin cytoskeleton
Inositol phosphate metabolism
Salmonella infection
NOD-like receptor signaling pathway
Endocytosis
Cellular senescence
Cytokine-cytokine receptor interaction
Tight junction
Metabolic pathways
Fold Enrichment
N. of Genes
-log10(FDR)
E
GO on segmentation timing devQTL
Lateral line nerve development
Embryonic axis specification
Anterior/posterior axis specification
Embryonic pattern specification
Mesoderm formation
Cranial nerve development
Mesoderm morphogenesis
Somitogenesis
Segmentation
Axis specification
Nerve development
Formation of primary germ layer
Peripheral nervous system development
Mesoderm development
Somite development
Anterior/posterior pattern specification
Extracellular matrix organization
Extracellular structure organization
External encapsulating structure organization
Anatomical structure formation involved in morphogenesis
Fold Enrichment
N. of Genes
-log10(FDR)
F
F'
Crispants of DE genes within QTL hits
mean period in minutes
intercept period in minutes
control Oca2 KO
adgrg3 KO
mmp15 KO
tgfbi KO
b4galt1 KO
G
mespb mis-sense variants Cab/Kaga
bp
a.a
S/P
A/G
E/D
H
pcdh10b mis-sense variants Cab/Kaga
C/T switch
T/C switch
A/T switch
ENSORLG00000020474.2 gene
ENSORLG00000020474.2 CDS
R/K
K/R
F/Y

◀ **Figure EV4. *dev*QTL mapping for segmentation timing and bulk-RNA sequencing on Kaga and Cab tails.**

(**A**) Workflow from *dev*QTL mapping to candidate gene selection. For segmentation timing 46,872 homozygous divergent SNPs between Kaga and Cab were located on chromosomes 3, 4 and 10. Genomic coordinates revealed a total of 57 genes on all 3 chromosomes located in regions that passed the significance threshold. Bulk RNA-sequencing on Kaga and Cab unsegmented PSM narrowed down the number of candidate genes expressed in the PSM to 35 genes. 29 of which were either differentially expressed or contained a coding consequence or both. GO enrichment and candidate gene picking led to a top 11 gene list which were selected to perform F0 CRISPR KOs in *her7-venus* Cab background to check for a clock period phenotype. (**B**) *ensembl* variant effect predictor (VEP) output for segmentation timing showing the distribution of homozygous divergent SNPs between Kaga and Cab in the F2 Kaga/Cab QTL mapping on segmentation timing, most of the divergent SNPs fall in either intronic or intergenic regions, of the ones that fall within the coding sequence of genes the majority lead to synonymous mutations (63.5%) while only a minority (35.9%) lead to miss-sense mutations. (**C**) Volcano plot showing differentially expressed (DE) genes on bulk RNA-sequencing of unsegmented Cab and Kaga tails at the 13–14 somite stage. 1431 genes are significantly downregulated in Cab compared to Kaga tails (blue), while 1175 genes are upregulated in Cab compared to Kaga tails (red). (**D**) Gene Ontology (GO) enrichment categories for upregulated and downregulated genes from the bulk RNA-Sequencing experiment shown in (**A**). (**E**) GO analysis on gene list from segmentation timing *dev*QTL mapping that were transcriptionally active in tail tissue (35 genes). (**F–F′**) endogenous *her7-venus* mean and intercept period analysis in F0 Cab Crispants imaged at the 10-11SS on genes differentially expressed and within the *dev*QTL peaks for segmentation timing. Kruskal–Wallis' test $P = 0.2$. (**F**) Kruskal–Wallis' test $P = 0.05$ (**F′**) $N = 11$ control CRISPR/Cas9 and *Oca2* injected Cab *her7-venus* embryos, $N = 14$ *adgrg3*, $N = 12$ *tgfbi*, $N = 13$ *mmp15*, $N = 29$ *b4galt1*, CRISPR/Cas9 injected into *Cab her7-venus*. (**G**) Position of mis-sense variants between Cab/Kaga in the coding sequence of *mespb*. Three base-pair (bp) changes cause 3 amino acid (a.a) changes: Serine/Proline (S/P), Alanine/Glycine (A/G) and Glutamic Acid/Aspartic Acid (E/D) are highlighted. Visualisation done using *Geneious*. (**H**) Position of mis-sense variants between Cab/Kaga in the coding sequence of *pcdh10b*. Three base-pair (bp) changes cause 3 amino acid (a.a) changes: Arginine/Lysine (R/K), Lysine/Arginine (K/R) and Phenylalanine/Tyrosine (F/Y) are highlighted. Visualisation using *Geneious*.

© The Author(s)

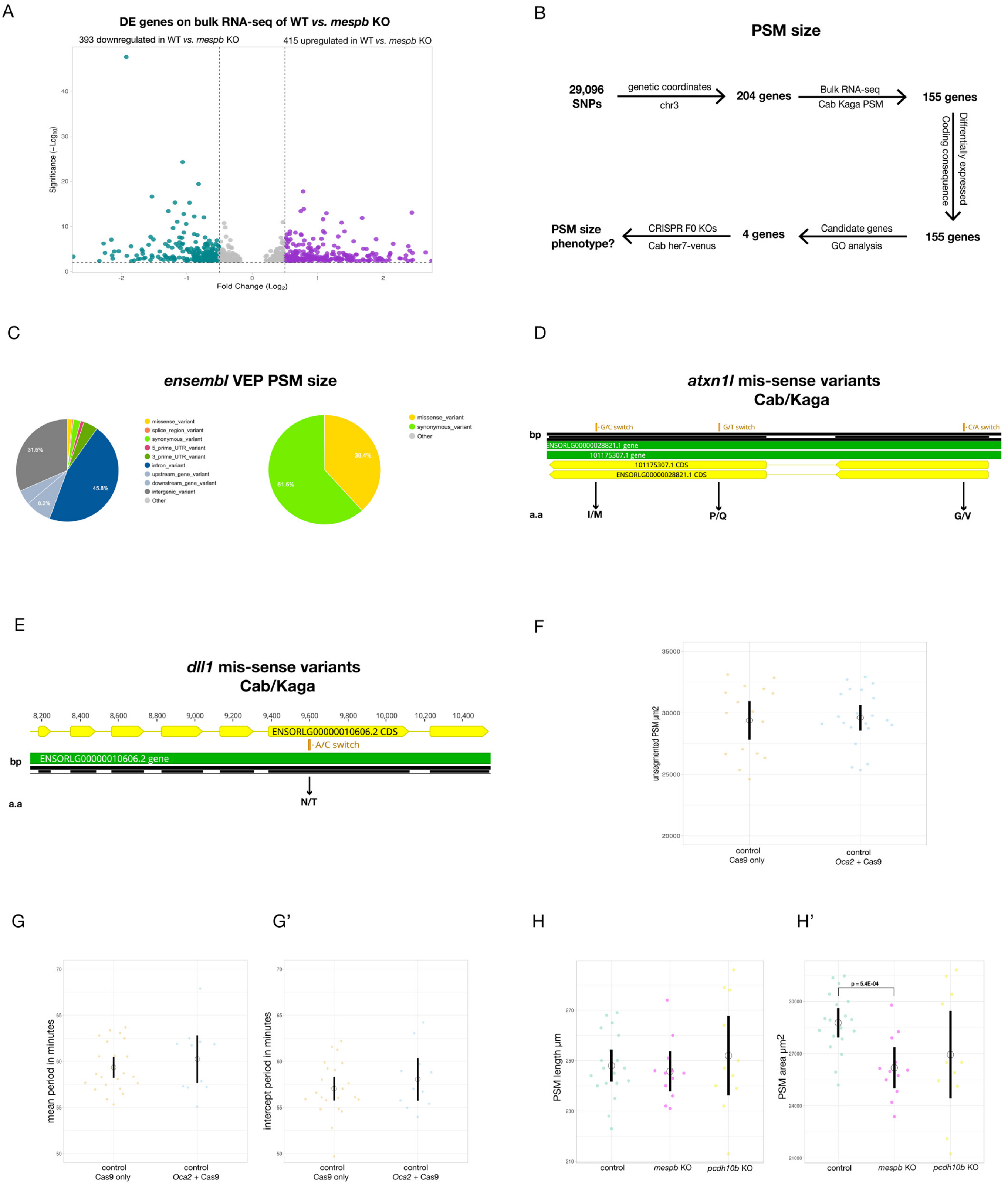
A
DE genes on bulk RNA-seq of WT vs. mespb KO
393 downregulated in WT vs. mespb KO
415 upregulated in WT vs. mespb KO
Significance (−Log10)
Fold Change (Log2)
B
PSM size
29,096 SNPs
genetic coordinates
chr3
204 genes
Bulk RNA-seq
Cab Kaga PSM
155 genes
Differentially expressed
Coding consequence
155 genes
Candidate genes
GO analysis
4 genes
CRISPR F0 KOs
Cab her7-venus
PSM size phenotype?
C
ensembl VEP PSM size
missense_variant
splice_region_variant
synonymous_variant
5_prime_UTR_variant
3_prime_UTR_variant
intron_variant
upstream_gene_variant
downstream_gene_variant
intergenic_variant
Other
31.5%
45.8%
8.2%
38.4%
61.5%
D
atxn1l mis-sense variants
Cab/Kaga
G/C switch
G/T switch
C/A switch
bp
ENSORLG00000028821.1 gene
101175307.1 gene
101175307.1 CDS
ENSORLG00000028821.1 CDS
a.a
I/M
P/Q
G/V
E
dll1 mis-sense variants
Cab/Kaga
ENSORLG00000010606.2 CDS
A/C switch
bp
ENSORLG00000010606.2 gene
a.a
N/T
F
unsegmented PSM μm2
control
Cas9 only
control
Oca2 + Cas9
G
mean period in minutes
control
Cas9 only
control
Oca2 + Cas9
G'
intercept period in minutes
control
Cas9 only
control
Oca2 + Cas9
H
PSM length μm
control
mespb KO
pcdh10b KO
H'
PSM area μm2
p = 5.4E-04
control
mespb KO
pcdh10b KO

Figure EV5. Bulk RNA-sequencing on Cab *wild-type* vs. *mespb* KO tails and *dev*QTL mapping on PSM size.

(**A**) Volcano plot showing differentially expressed genes on bulk RNA-sequencing of Cab *wild-type* and *mespb* Crispant tails at the 13–14 somite stage. 393 genes are significantly downregulated in *wild-type* compared to *mespb* Crispant tails (green), while 415 genes are upregulated in *wild-type* compared to *mespb* Crispant (magenta). (**B**) Workflow from *dev*QTL mapping to candidate gene selection. For PSM size 29,096 homozygous divergent SNPs between Kaga and Cab are located on chromosomes 3. Genomic coordinates revealed a total of 204 genes located in regions that passed the significance threshold. Bulk RNA-sequencing on Kaga and Cab unsegmented PSM showed 155 genes transcriptionally acitve. GO annotation and candidate gene picking led to a top 4 genes which were selected to perform F0 CRISPR/Cas9 KOs in Cab background to assess PSM size. (**C**) VEP output for PSM size showing the distribution of homozygous divergent SNPs between Kaga and Cab in the F2 Kaga/Cab QTL on PSM size, majority of the divergent SNPs fall in either intronic or intergenic regions, of the ones that fall within the coding sequence of genes the majority lead to synonymous mutations (61.5%) while only a minority (38.4%) lead to miss-sense mutations. (**D**) position of mis-sense variants between Cab/Kaga in the coding sequence of *atxn1l*. Three base-pair (bp) changes cause 3 amino acid (a.a) changes: Isoleucine/Methionine (I/M), Proline/Glutamine (P/Q) and Glycine/Valine (G/V) are highlighted. Visualization using *Geneious*. (**E**) position of mis-sense variants between Cab/Kaga in the coding sequence of *dll1*. One base-pair (bp) change causes 1 amino acid (a.a) change: Asparagine/Threonine (N/T) is highlighted. Visualisation using *Geneious*. (**F**) Comparison of PSM size for *Oca2* + Cas9 injected control embryos as opposed to Cas9 only injected control embryos (shown in Fig. 4). Welch two sample *t* test $P = 0.80$. $N = 11$ *Oca2* + Ca9, $N = 23$ Cas9 only (**G–G′**) Comparison of endogenous *her7-venus* mean and intercept period values for *Oca2* + Cas9 injected control embryos (shown in EV4F-F′) as opposed to Cas9 only injected control embryos (shown in Fig. 4; Fig. S4A,B) Welch two sample *t* test $P = 0.48$ (**G**) and $P = 0.39$ (**G′**) $N = 11$ *Oca2* + Ca9, $N = 23$ Cas9 only. (**H–H′**) PSM size in *mespb* and *pcdh10b* Crispants compared to control embryos. Welch two sample *t* test PSM length *mespb* KO $P = 0.60$, *pcdh10b* KO $P = 0.59$. Welch two sample *t* test PSM area *mespb* KO $P = 5E-04$, *pcdh10b* KO $P = 0.13$. $N = 19$ control embryos, $N = 13$ *mespb* KO Crispants, $N = 11$ *pcdh10b* KO Crispants.

 © The Author(s)
